# PP2A and tumor radiotherapy

**DOI:** 10.1186/s41065-020-00149-7

**Published:** 2020-08-26

**Authors:** Xiao Lei, Na Ma, Lehui Du, Yanjie Liang, Pei Zhang, Yanan Han, Baolin Qu

**Affiliations:** The First Medical Center of Chinese PLA General Hospital, Department of Radiation Oncology, Beijing, P. R. China

**Keywords:** Protein phosphatase 2A, Conventional tumor radiotherapy, DNA damage response, Radiosensitization effects

## Abstract

Protein phosphatase 2A (PP2A) is a serine/threonine phosphatase that serves as a key regulator of cellular physiology in the context of apoptosis, mitosis, and DNA damage responses. Canonically, PP2A functions as a tumor suppressor gene. However, recent evidence suggests that inhibiting PP2A activity in tumor cells may represent a viable approach to enhancing tumor sensitivity to chemoradiotherapy as such inhibition can cause cells to enter a disordered mitotic state that renders them more susceptible to cell death. Indeed, there is evidence that inhibiting PP2A can slow tumor growth following radiotherapy in a range of cancer types including ovarian cancer, liver cancer, malignant glioma, pancreatic cancer, and nasopharyngeal carcinoma. In the present review, we discuss current understanding of the role of PP2A in tumor radiotherapy and the potential mechanisms whereby it may influence this process.

## Introduction

While mainstays of tumor treatment efforts, conventional radiotherapy and chemotherapy often yield unsatisfactory therapeutic outcomes [1–3]. These poor outcomes are generally linked to tumor cell multidrug resistance and resistance to ionizing radiation [4–6]. In addition, while these treatments are well-tailored to killing rapidly proliferating tumor cells, they generally fail to impact hypoxic and quiescent cells, ultimately resulting in treatment failure and tumor recurrence [7–9]. Understanding the mechanistic basis for tumor cell chemoresistance and radioresistance is thus vital. Interestingly, recent research evidence suggests that radiosensitization can be achieved by accelerating cell cycle progression in quiescent cells such that they become proliferative [10, 11]. Inhibiting proteins such as PP2A can drive quiescent tumor cells to enter mitosis, in turn potentially increasing tumor cell sensitivity to treatment [12, 13]. Inhibiting PP2A may therefore represent a valuable new approach to promoting tumor radiosensitization. In the present review, we discuss current research progress pertaining to the role of PP2A in the context of tumor radiotherapy.

### The role of PP2A in radiation therapy

PP2A is a serine/threonine phosphatase that functions as a tumor suppressor gene [14]. It is a complex composed of a core enzyme and a regulatory subunit. The core enzyme (PP2AD) is a dimer comprised of a 36 kD catalytic subunit (PP2A c) and a 65 kD regulatory subunit (PR65 or subunit A). PP2A has three subunits, including subunit A and two subtypes of subunit C (α and β), with each of these subunits exhibiting distinct structural and catalytic activities. There are also multiple subtypes of subunit B that serve to control the specificity and localization of PP2A. Overall, there are four families of regulatory B subunits capable of binding to the core enzyme: B (PR55), B′ (B56 or PR61), B“ (PR72), and B’“ (PR93/PR110) [15–17]. Early research suggested that PP2A functions as a classic tumor suppressor gene that is downregulated or nonfunctional in many tumor types including lung, skin, breast, brain, ovarian, cervical, and colon cancers [18–20]. At a functional level, PP2A inhibits a range of tumor signaling pathways [21], preventing IL-2-induced JAK3 and STAT5 activation, which is normally dysregulated in many malignancies [22]. PP2A can also interact with the ERK2/MEK and Ras/Raf signaling pathways through direct and indirect mechanisms so as to control their activation. Given that constitutive Ras/Raf/MEK/ERK signaling is a characteristic of many malignant tumor cells [23–26], these highlights another mechanism whereby PP2A can control oncogenesis. PP2A can also mediate proteasome dephosphorization and thereby impact c-Myc, which is often constitutively active in the context of tumorigenic transformation [27, 28].

Tumor metastasis, recurrence, and radioresistance all represent major roadblocks to the effective treatment of cancer patients [29, 30]. Following PP2A inhibition, many tumors exhibit slower growth, increased apoptotic cell death, and greater sensitivity to ionizing radiation, as has been observed in the context of nasopharyngeal carcinoma, ovarian cancer, pancreatic cancer, liver cancer, and malignant glioma [31–35]. In malignant glioma, for example, PP2A inhibition increases the frequency of cells in the M phase of mitosis, inhibiting tumor proliferation while driving increased radiosensitivity [31]. Similarly, PP2A inhibition in nasopharyngeal carcinoma has been linked to significant increases in the frequency of apoptotic cells and G2/M arrest [36]. Likewise, inhibiting PP2A in cancer significantly delayed DMA damage repair and thereby facilitated more rapid cell death following irradiation [37].

### Potential mechanisms whereby PP2A influences radiotherapy outcomes

#### The role of PP2A in mitosis

PP2A is a key regulator of normal mitotic processes [38]. Greatwall kinase inhibited PP2A by small proteins ENSA and ARPP19, thereby attenuating PP2A-regulated Cdk1 dephosphorylation and promoting mitosis, whereas severe mitotic defects occur in the absence of greatwall kinase [39, 40]. PP2A also negatively regulates Cdk1 activity via activating wee1/myt1 and by inhibiting cdc25 [41]. Inhibiting PP2A can also drive the upregulation of molecules downstream of Cdk1, thereby promoting mitosis. This greatwall kinase/PP2A signaling pathway is thought to be a primary regulator of normal Cdk1 functionality in the context of mitosis [42]. PP2A can also act on other mitotic mediators such as the mitosis-specific kinases PLK1, which is a key marker of G2/M phase arrest following PP2A inhibition and which interacts with centrosomes during mitosis [43, 44]. PP2A is also involved in the negative feedback inhibition of PLK1 and Aurora B, thereby regulating the spindle collection checkpoint in order to ensure that microtubules are properly connected to the centromere [45].

#### Inhibiting PP2A causes G2/M cell cycle checkpoint inactivation and alters DNA damage repair

Radiation-induced DNA damage can induce cell cycle arrest and DNA damage repair that is mediated by DNA damage checkpoint activation [46]. Irradiation-associated DNA damage can lead to G2/M checkpoint activation and consequent G2/M phase arrest, enabling DNA repair to occur prior to cellular entry into mitosis [47]. Cdc2/Cyclin B is a key regulator of this G2/M checkpoint, as Cdc2/Cyclin B activation is required in order for cells to proceed from the G2 phase into the cleavage phase [48]. DNA damage is rapidly followed by the phosphorylation and activation of the ATR and ATM kinases, which in turn activate Chk2 and Chk1 [49]. Chk2 and Chk1 function in part by suppressing the activation of Cdc25 family proteins such that Cdc2/Cyclin B activation is inhibited. Following DNA damage, Cdc2/Cyclin B activity is thus reduced, resulting in cell cycle arrest [50]. Following drug- or radiation-induced DNA damage, PP2A dephosphorylation can inhibit PLK1, which phosphorylates and activates Cdc25 and cyclins involved in the G2/M checkpoint, thereby facilitating cell cycle progression. PP2A may thus prevent cells from dividing by inhibiting PLK1 [44]. Moreover, inhibition of PP2A showed that radiation-induced inactivation of ATR and Chk1 kinase, phosphorylation of Cdc2-Tyr15, and inactivation of G2/M phase checkpoints, which attenuated radiation-induced G2/M arrest, thereby enabling tumor cells to enter into mitosis via reducing DNA damage repair efficiency and aggravating cellular mitotic disorders [51].

#### Inhibiting PP2A promotes G0 stage tumor cell entry into mitosis

Cdk2 activity has recently been found to govern the proliferation of quiescent cells following mitosis, such that cells enter the G0 phase when Cdk2 activity levels are low. Regulating Cyclin E/Cdk2 activity at the end of the cell cycle can promote cellular proliferation [12]. In adult organisms, PP2A has been found to promote cellular quiescence [52]. In studies of Drosophila eyes and wings, researchers have determined that inhibiting PP2A at the end of the cell cycle can induce additional cell division and thereby impair such quiescence. In these Drosophila, the PP2A subunit B56 family member wdb serves as an important regulator of PP2A-related cellular quiescence. When PP2A activity is suppressed, cells that would normally enter the stationary phase instead exhibit robust Cdk2 activity [53]. Ectopic dominant testing has further revealed that abnormal Cyclin E/Cdk2 activity can promote additional cell cycle progression in the context of PP2A inhibition [12]. Reduced wdb/PP2A activity results in abnormally elevated Cyclin E levels, enabling quiescent cells to pass through the G0 phase and to thereby enter into mitosis [54], increasing cellular sensitivity to radiotherapy and chemotherapy. Given the important role of tumor cell quiescence as a driver of tumor radioresistance and recurrence in cancer patients [55], inhibiting PP2A may represent a viable means of promoting tumor radiosensitivity by driving cells in the G0 phase of the cell cycle to undergo mitosis.

#### PP2A as a regulator of apoptosis

PP2A can control apoptosis by influencing both PI3K/Akt pathway signaling and the expression and activity of apoptosis-associated proteins [56]. In cells with functional Bcl-2, for example, PP2A has been shown to promote Bcl-2 dephosphorization and to thereby promote apoptotic cell death [57–59]. In contrast, in cells that are highly metabolically active, PP2A can dephosphorylate and thereby activate CaMKII so as to exert an anti-apoptotic effect [60]. PP2A also modulates the P53 pathway such that it can activate Bax/Noxa/Puma and inhibit Bcl-2 to drive apoptotic death [61–63].

In the context of the DNA damage response, the ATM signaling pathway directly activates and stabilizes PP2A by phosphorylating the ubiquitin ligase MDM2. PP2A in turn inhibits Akt1 pathway activity and thereby suppresses MDM2 activation, thus preventing the MDM2-mediated degradation of p53 [64]. In the presence of irreversible DNA damage, PP2A can also directly dephosphorylate p53, stabilizing this protein an inducing cell cycle arrest and apoptosis [65]. Inhibiting PP2A may therefore be a viable therapeutic strategy in highly metabolically active tumor cells. Suppressing PP2A activity in cells exhibiting DNA damage can also inhibit Bax expression and promote the cell cycle [65]. Studies of combination radiotherapy and PP2A inhibition have highlighted the consequent inhibition of interactions between p53 and PP2A, reducing the role of the p53 pathway in response to DNA damage and promoting cellular proliferation and p53-independent apoptotic cell death [66].

#### PP2A as a regulator of the WNT/β-catenin signaling pathway

PP2A is capable of inhibiting WNT/β-catenin signaling pathway activity [67], which normally plays important roles in governing the migration and proliferation of cells [68]. After WNT ligands interact with specific cell surface receptors, the Tcf family transcriptional coactivator β-catenin undergoes nuclear translocation, interacts with Tcf, and modulates target gene expression. This process often becomes constitutively activated during the early stages of oncogenesis [69]. In tumor cells in which the WNT signaling pathway is not active, cytoplasmic β-catenin is generally degraded. A complex composed of APC, DVL, Axin, and β-3-glycogen synthesis kinase can target β-catenin for degradation [31]. However, the PP2A-C regulatory subunit has also been shown to play downstream signaling roles in the context of the WNT/β-catenin signaling pathway [70]. Aspirin has also been found to downregulate WNT/β-catenin signaling pathway activity via inhibiting PP2A [71]. Positive PP2A feedback signaling has also been suggested to alter the WNT/β-catenin signaling pathway in pancreatic cancer and colorectal cancer cell lines, thereby stabilizing the activation of this pathway [72, 73].

### Current clinical approaches to inhibiting PP2A as an approach to tumor radiosensitization

To date, pharmacological inhibition of PP2A has largely been dependent upon the use of natural compounds such as okadaic acid and anthraquinone [74]. These compounds, however, exhibit varying degrees of toxicity. In contrast, LB100 is a water-soluble PP2A inhibitor that is less toxic than these other compounds. Research suggests that while radiotherapy can enhance PP2A activity, LB100 pretreatment prior to radiotherapy can suppress PP2A activation while simultaneously enhancing tumor sensitivity to irradiation [75]. LB100 has been leveraged in several clinical trials as a PP2A inhibitor owing to its efficacy and low toxicity [76]. In one study of pancreatic cancer, for example, LB100 was found to effectively radiosensitize pancreatic cancer cells without adversely affecting normal small intestinal cells [77]. LB100-mediated PP2A inhibition has also been shown to prevent radiation-induced Rad51 foci formation and homologous recombination repair, thereby causing sustained DNA damage in cells following radiation exposure [77]. The presence of undifferentiated stem-like tumor cells capable of undergoing self-renewal is thought to be one of the key mechanisms underlying tumor recurrence and therapeutic resistance. Traditional radiotherapy and chemotherapy efforts are largely unable to impact these cancer step cells, as they grow slowly and are largely quiescent [78, 79]. There is recent experimental evidence that the receptor co-repressor protein complex is a primary determinant of the stem-like properties of cancer stem cells in glioma tumors [80]. This receptor co-repressor protein complex is composed of the receptor co-repressor protein, a deacetylase complex, steroids, hormone receptors, and transcription factors that function to control transcription in the context of glial differentiation [81]. Cytokine-induced ciliary neurotrophic factor stimulation of glioma precursor cells has been shown to inhibit receptor co-repressor protein complex activity via Akt/PI3K-mediated phosphorylation of the receptor co-repressor protein [82]. Inhibition of PP2A using LB100 resulted in enhanced Ak1 activity, thereby preventing receptor co-repressor protein complex formation and promoting cellular division, rendering quiescent tumor cells more sensitive to irradiation [31].

### Perspectives

Inhibiting PP2A has been conclusively shown to enhance tumor cell radiosensitivity. However, further research is necessary in order to facilitate the optimal clinical implementation of these experimental findings. For example, while many studies have assessed the impact of inhibiting PP2A in tumor cells following radiation exposure, few studies have assessed the effect of such inhibition on normal tissues, which may also undergo potential radiosensitization [83–85]. Differences in PP2A expression profiles between normal and tumor tissues are also essential to ensure that tumor cells can be effectively killed without causing undue harm to healthy tissues. At present, there are also few specific inhibitors of PP2A available. To leverage the potential clinical utility of combination PP2A inhibition and radiotherapy treatment, it is vital that novel highly specific PP2A inhibitors be developed. The identification of specific inhibitors that preferentially target tumor cells while leaving healthy cells intact would further advance the clinical applications of PP2A inhibition. It is also important to note that many studies of PP2A inhibition have focused only on single factors [86–88], whereas tumor resistance and recurrence are multifactorial in nature. At present, there is a dearth of systematic or comprehensive studies pertaining to the mechanisms whereby PP2A inhibition bolsters the efficacy of radiation therapy.

## Conclusion

In summary, inhibiting PP2A in combination with radiotherapeutic treatment may represent a viable approach to enhancing patient treatment outcomes and preventing tumor recurrence. However, further research regarding the mechanisms underlying such combination efficacy is still required. In addition, more specific pharmacological inhibitors of PP2A must be developed in order to achieve better clinical outcomes.

## Data Availability

The datasets are available under reasonable request.

## Abbreviations

PP2A: Protein phosphatase 2A
Cdk1: Cyclin Dependent Kinase 1
PLK1: polo-like kinase 1
Cdc2: Cyclin Dependent Kinase 2
ATM: Ataxia telangiectasia mutated
ATR: ATM and RAD3-related
PI3K: Phosphoinositide 3-kinase
MDM2: Murine double minute-2
DNA: Deoxyribonucleic Acid
RNA: Ribonucleic Acid
